# 2,2,3,3,4,4,5,5-Octa­fluorohexa­ne-1,6-diol

**DOI:** 10.1107/S2414314620004459

**Published:** 2020-04-07

**Authors:** Kylie Feightner, Douglas R. Powell, Christopher M. Burba

**Affiliations:** a Department of Natural Sciences, Northeastern State University, 611 N. Grand Ave., Tahlequah, OK 74464, USA; b Department of Chemistry and Biochemistry, University of Oklahoma, 101 Stephenson Parkway, Norman, OK 73019, USA; University of Toronto, Canada

**Keywords:** crystal structure, fluorinated glycol, hydrogen bonding

## Abstract

In the crystal of the title compound, C_6_H_6_F_8_O_4_, O—H⋯O hydrogen bonds involving the hy­droxy groups connect the mol­ecules, forming a two-dimensional network parallel to (100). These hydrogen-bonding inter­actions appear to drive the O—C—C—O torsion angles into a *gauche*–*trans*–*trans* series of conformations along the backbone of the mol­ecule.

## Abstract 2

The title compound exhibits *gauche*–*trans*–*trans* O—C—C—O conformations. The conformational configuration is driven by the formation of an extended hydrogen-bonding network among the terminal alcohol groups of each mol­ecule.

## Graphical abstract

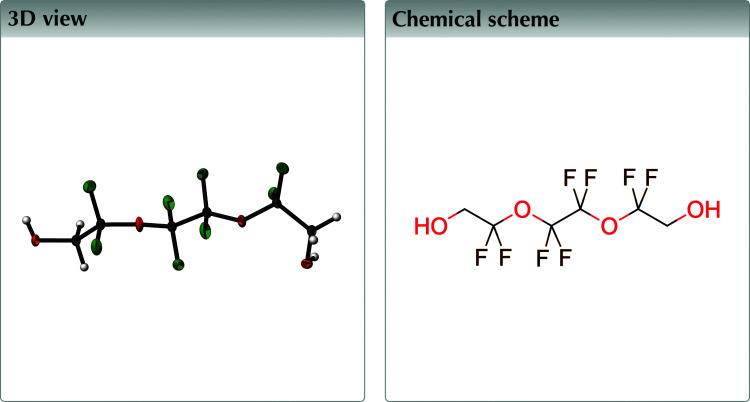

## Structure description

**Figure 1:**
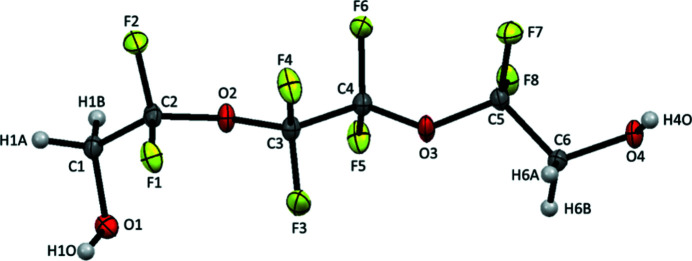
The mol­ecular structure of the title compound. Displacement ellipsoids are shown at 50% probability level.

**Figure 2:**
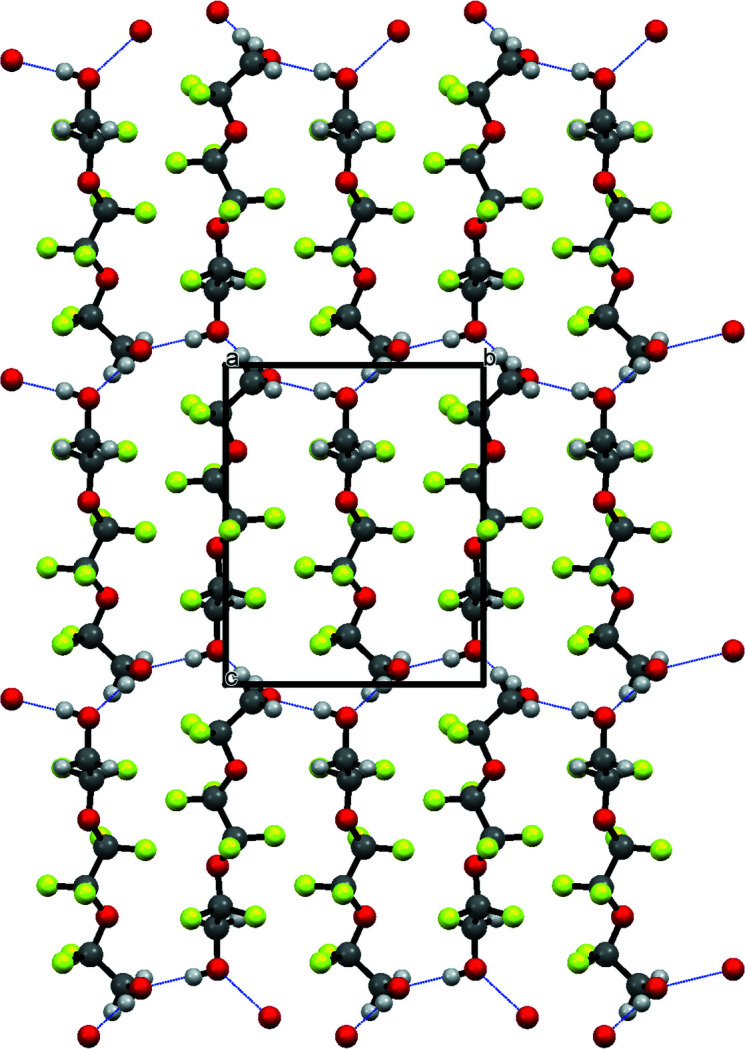
Part of the crystal structure with hydrogen bonds shown as dashed lines.

Ionic liquids have attracted considerable inter­est as solvents for a variety of applications. ‘Solvate’ ionic liquids (SILs) are a new class of ionic liquids that consist of equimolar mixtures of inorganic salts and mol­ecular solvents capable of chelating the cations of the salt (Ueno *et al.*, 2012 ▸, 2015 ▸; Mandai *et al.*, 2014 ▸, 2015 ▸). Most research on SILs focus on methyl-capped ethyl­ene oxide mol­ecular solvents, which are collectively known as ‘glymes’. Structural variation of the chelating compound will undoubtedly influence cation–solvent inter­actions and provide alternative means for tuning SIL properties (Saito *et al.*, 2016 ▸). Our lab has pursued this line of research by examining partially fluorinated mol­ecular solvents for SIL applications. During our experiments, we isolated and determined the structure of the title compound, a partially fluorinated derivative of tri­ethyl­ene glycol. The mol­ecular structure of the title compound is shown in Fig. 1 ▸. In the crystal, O—H⋯O hydrogen bonds involving the terminal hydroxyl groups (see Table 1 ▸) connect the mol­ecules, forming a two-dimensional network parallel to (100) (Fig. 2 ▸). In addition, a weak inter­molecular C—H⋯F hydrogen bond is observed within this network. These hydrogen-bonding inter­actions appear to drive the O—C—C—O torsion angles into a *gauche*–*trans*–*trans* series of conformations along the backbone of the mol­ecule: O1—C1—C2—O2 = 66.3 (2), O2—C3—C4—O2 = −168.91 (15), and O3—C5—C6—O4 = −177.92 (15)°. By way of comparison, the O—C—C—O torsion angles are *gauche* in monoglyme (Yoshihiro *et al.*, 1996 ▸) and longer chain glymes (Johansson *et al.*, 2010 ▸; Hyun *et al.*, 2001 ▸; Tadokoro, 1964 ▸).

## Synthesis and crystallization

2,2,3,3,4,4,5,5-Octa­fluoro-1,6-hexa­nediol (1.94 mmol) was added to a 1:1 molar ratio mixture of lithium bis­(tri­fluoro­methane­sulfon­yl)imide (2.3 mmol) and 2,2′-[ethane-1,2-diylbis(­oxy)]di(ethan-1-ol) (commonly known as tri­ethyl­ene glycol; 2.3 mmol). The resulting mixture was stirred at 353 K for 6 h to produce a homogenous, viscous solution. Colorless, plate-shaped single crystals formed from the solution upon standing over a period of days.

## Refinement

Crystal data, data collection methods, and structural refinement details are provided in Table 2 ▸. The absolute structure of the title compound could not be established in the refinement reported here.

## Supplementary Material

Crystal structure: contains datablock(s) I. DOI: 10.1107/S2414314620004459/lh4054sup1.cif

Structure factors: contains datablock(s) I. DOI: 10.1107/S2414314620004459/lh4054Isup2.hkl

Supporting information file. DOI: 10.1107/S2414314620004459/lh4054Isup3.cml

CCDC reference: 1993931

Additional supporting information:  crystallographic information; 3D view; checkCIF report

## Figures and Tables

**Table 1:** Hydrogen-bond geometry (Å, °)

*D*—H⋯*A*	*D*—H	H⋯*A*	*D*⋯*A*	*D*—H⋯*A*
O1—H1*O*⋯O4^i^	0.84 (4)	1.87 (4)	2.705 (2)	172 (3)
O4—H4*O*⋯O1^ii^	0.85 (4)	1.82 (4)	2.661 (2)	173 (3)
C6—H6*B*⋯F4^iii^	0.99	2.52	3.416 (2)	151

Symmetry codes: (i) 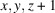 ; (ii) 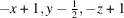 ; (iii) 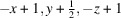 .

**Table 2:** Experimental details

Crystal data	
Chemical formula	C_6_H_6_F_8_O_4_
*M*_r_	294.11
Crystal system, space group	Monoclinic, *P*2_1_
Temperature (K)	100
*a*, *b*, *c* (Å)	5.3009 (8), 8.6250 (12), 10.6976 (14)
β (°)	91.146 (5)
*V* (Å^3^)	489.00 (12)
*Z*	2
Radiation type	Mo *K*α
μ (mm^−1^)	0.25
Crystal size (mm)	0.49 × 0.49 × 0.05

Data collection	
Diffractometer	Bruker Photon II CMOS
Absorption correction	Multi-scan (*SADABS*; Krause *et al.*, 2015 ▸)
*T*_min_, *T*_max_	0.543, 0.746
No. of measured, independent and observed [*I* > 2σ(*I*)] reflections	14748, 2985, 2896
*R*_int_	0.049
(sin θ/λ)_max_ (Å^−1^)	0.715

Refinement	
*R*[*F* ^2^ > 2σ(*F* ^2^)], *wR*(*F* ^2^), *S*	0.038, 0.100, 1.00
No. of reflections	2985
No. of parameters	170
No. of restraints	1
H-atom treatment	H atoms treated by a mixture of independent and constrained refinement
Δρ_max_, Δρ_min_ (e Å^−3^)	0.65, −0.40

Computer programs: *APEX3* (Bruker, 2018 ▸), *SAINT* (Bruker, 2016 ▸), *SHELXT* (Sheldrick, 2015*a* ▸), *SHELXL2018/3* (Sheldrick, 2015*b* ▸) and *Mercury* (Macrae *et al.*, 2020 ▸).

## full crystallographic data

### Crystal data

**Table:**

C_6_H_6_F_8_O_4_	*F*(000) = 292
*M**_r_* = 294.11	*D*_x_ = 1.997 Mg m^−^^3^
Monoclinic, *P*2_1_	Mo *K*α radiation, λ = 0.71073 Å
*a* = 5.3009 (8) Å	Cell parameters from 9936 reflections
*b* = 8.6250 (12) Å	θ = 3.0–30.5°
*c* = 10.6976 (14) Å	µ = 0.25 mm^−^^1^
β = 91.146 (5)°	*T* = 100 K
*V* = 489.00 (12) Å^3^	Plate, colourless
*Z* = 2	0.49 × 0.49 × 0.05 mm

### Data collection

**Table:**

Bruker Photon II CMOS diffractometer	2896 reflections with *I* > 2σ(*I*)
Radiation source: microfocus sealed tube	*R*_int_ = 0.049
ω and φ scans	θ_max_ = 30.5°, θ_min_ = 3.0°
Absorption correction: multi-scan (SADABS; Krause *et al.*, 2015)	*h* = −7→7
*T*_min_ = 0.543, *T*_max_ = 0.746	*k* = −12→12
14748 measured reflections	*l* = −15→15
2985 independent reflections	

### Refinement

**Table:**

Refinement on *F*^2^	Secondary atom site location: difference Fourier map
Least-squares matrix: full	Hydrogen site location: mixed
*R*[*F*^2^ > 2σ(*F*^2^)] = 0.038	H atoms treated by a mixture of independent and constrained refinement
*wR*(*F*^2^) = 0.100	*w* = 1/[σ^2^(*F*_o_^2^) + (0.075*P*)^2^ + 0.078*P*] where *P* = (*F*_o_^2^ + 2*F*_c_^2^)/3
*S* = 1.00	(Δ/σ)_max_ < 0.001
2985 reflections	Δρ_max_ = 0.65 e Å^−^^3^
170 parameters	Δρ_min_ = −0.40 e Å^−^^3^
1 restraint	Absolute structure: Refined as an inversion twin
Primary atom site location: dual	Absolute structure parameter: 0.5 (6)

### Special details

**Table:**

Geometry. All esds (except the esd in the dihedral angle between two l.s. planes) are estimated using the full covariance matrix. The cell esds are taken into account individually in the estimation of esds in distances, angles and torsion angles; correlations between esds in cell parameters are only used when they are defined by crystal symmetry. An approximate (isotropic) treatment of cell esds is used for estimating esds involving l.s. planes.
Refinement. Refined as a 2-component inversion twin.

### Fractional atomic coordinates and isotropic or equivalent isotropic displacement parameters (Å^2^)

**Table:**

	*x*	*y*	*z*	*U*_iso_*/*U*_eq_	
F1	0.7033 (3)	0.36867 (16)	0.87486 (11)	0.0193 (3)	
F2	1.1035 (3)	0.40107 (18)	0.84955 (14)	0.0238 (3)	
F3	0.4638 (2)	0.4558 (2)	0.65888 (13)	0.0241 (3)	
F4	0.7907 (3)	0.31004 (16)	0.63351 (13)	0.0232 (3)	
F5	0.7219 (3)	0.68928 (15)	0.52189 (12)	0.0214 (3)	
F6	1.0050 (2)	0.51638 (17)	0.48334 (12)	0.0192 (3)	
F7	0.7953 (2)	0.36716 (19)	0.26323 (13)	0.0225 (3)	
F8	0.7493 (3)	0.61560 (17)	0.26968 (12)	0.0204 (3)	
O1	0.6422 (3)	0.67095 (18)	0.95429 (14)	0.0166 (3)	
H1O	0.556 (6)	0.614 (5)	1.001 (3)	0.020*	
O2	0.8372 (3)	0.54080 (17)	0.73056 (13)	0.0155 (3)	
O3	0.5994 (3)	0.46959 (19)	0.42741 (13)	0.0167 (3)	
O4	0.4017 (3)	0.46960 (18)	0.10482 (13)	0.0155 (3)	
H4O	0.395 (6)	0.376 (5)	0.081 (3)	0.019*	
C1	0.8841 (4)	0.6040 (2)	0.94631 (18)	0.0155 (3)	
H1A	0.935916	0.560859	1.028644	0.019*	
H1B	1.007975	0.684471	0.923235	0.019*	
C2	0.8810 (3)	0.4762 (2)	0.84895 (17)	0.0144 (3)	
C3	0.7124 (3)	0.4577 (2)	0.63955 (17)	0.0137 (3)	
C4	0.7627 (3)	0.5366 (2)	0.51257 (17)	0.0130 (3)	
C5	0.6362 (3)	0.4807 (2)	0.29963 (17)	0.0133 (3)	
C6	0.3799 (3)	0.4651 (2)	0.23611 (17)	0.0138 (3)	
H6A	0.301564	0.365806	0.260933	0.017*	
H6B	0.269087	0.550509	0.263425	0.017*	

### Atomic displacement parameters (Å^2^)

**Table:**

	*U*^11^	*U*^22^	*U*^33^	*U*^12^	*U*^13^	*U*^23^
F1	0.0299 (6)	0.0142 (5)	0.0140 (6)	−0.0041 (5)	0.0021 (5)	0.0020 (4)
F2	0.0236 (6)	0.0300 (7)	0.0180 (6)	0.0135 (5)	−0.0003 (5)	0.0004 (5)
F3	0.0166 (5)	0.0397 (8)	0.0161 (5)	−0.0061 (6)	0.0016 (4)	0.0022 (6)
F4	0.0426 (8)	0.0115 (5)	0.0154 (6)	0.0009 (5)	−0.0001 (5)	0.0005 (4)
F5	0.0357 (7)	0.0126 (6)	0.0158 (6)	0.0026 (5)	−0.0025 (5)	0.0015 (5)
F6	0.0138 (5)	0.0295 (7)	0.0145 (5)	−0.0016 (4)	0.0008 (4)	0.0025 (5)
F7	0.0183 (5)	0.0271 (7)	0.0221 (6)	0.0067 (5)	−0.0018 (5)	−0.0056 (6)
F8	0.0256 (6)	0.0218 (6)	0.0139 (5)	−0.0114 (5)	−0.0010 (4)	0.0020 (5)
O1	0.0205 (6)	0.0135 (6)	0.0157 (6)	0.0030 (5)	0.0022 (5)	0.0021 (5)
O2	0.0229 (6)	0.0139 (6)	0.0095 (6)	−0.0027 (5)	−0.0031 (5)	0.0014 (5)
O3	0.0181 (6)	0.0235 (7)	0.0085 (5)	−0.0073 (6)	−0.0021 (4)	0.0004 (6)
O4	0.0224 (6)	0.0143 (6)	0.0097 (6)	−0.0018 (5)	−0.0029 (5)	−0.0011 (5)
C1	0.0178 (8)	0.0169 (8)	0.0116 (7)	0.0003 (6)	−0.0025 (6)	−0.0014 (6)
C2	0.0180 (7)	0.0147 (8)	0.0105 (7)	0.0019 (7)	−0.0017 (6)	0.0021 (6)
C3	0.0169 (7)	0.0132 (8)	0.0111 (7)	−0.0024 (6)	−0.0010 (6)	0.0011 (6)
C4	0.0149 (7)	0.0134 (8)	0.0108 (7)	−0.0009 (6)	−0.0008 (6)	0.0005 (6)
C5	0.0149 (7)	0.0151 (8)	0.0101 (7)	−0.0025 (6)	0.0004 (6)	−0.0007 (6)
C6	0.0136 (6)	0.0163 (8)	0.0116 (7)	−0.0007 (7)	−0.0015 (5)	−0.0010 (6)

### Geometric parameters (Å, º)

**Table:**

F1—C2	1.354 (2)	O3—C4	1.372 (2)
F2—C2	1.345 (2)	O3—C5	1.388 (2)
F3—C3	1.338 (2)	O4—C6	1.412 (2)
F4—C3	1.342 (2)	O4—H4O	0.85 (4)
F5—C4	1.338 (2)	C1—C2	1.517 (3)
F6—C4	1.339 (2)	C1—H1A	0.9900
F7—C5	1.354 (2)	C1—H1B	0.9900
F8—C5	1.351 (2)	C3—C4	1.547 (3)
O1—C1	1.410 (2)	C5—C6	1.513 (2)
O1—H1O	0.84 (4)	C6—H6A	0.9900
O2—C3	1.368 (2)	C6—H6B	0.9900
O2—C2	1.399 (2)		
			
C1—O1—H1O	108 (2)	F4—C3—C4	108.44 (15)
C3—O2—C2	120.31 (16)	O2—C3—C4	107.76 (16)
C4—O3—C5	121.70 (15)	F5—C4—F6	107.64 (16)
C6—O4—H4O	106 (2)	F5—C4—O3	111.28 (16)
O1—C1—C2	109.99 (15)	F6—C4—O3	112.68 (16)
O1—C1—H1A	109.7	F5—C4—C3	109.64 (16)
C2—C1—H1A	109.7	F6—C4—C3	109.31 (15)
O1—C1—H1B	109.7	O3—C4—C3	106.26 (15)
C2—C1—H1B	109.7	F8—C5—F7	105.85 (15)
H1A—C1—H1B	108.2	F8—C5—O3	111.40 (15)
F2—C2—F1	106.43 (17)	F7—C5—O3	109.52 (16)
F2—C2—O2	109.02 (16)	F8—C5—C6	111.64 (16)
F1—C2—O2	110.76 (15)	F7—C5—C6	111.42 (16)
F2—C2—C1	110.45 (15)	O3—C5—C6	107.05 (14)
F1—C2—C1	110.80 (16)	O4—C6—C5	110.69 (14)
O2—C2—C1	109.34 (16)	O4—C6—H6A	109.5
F3—C3—F4	107.59 (17)	C5—C6—H6A	109.5
F3—C3—O2	111.10 (16)	O4—C6—H6B	109.5
F4—C3—O2	112.68 (16)	C5—C6—H6B	109.5
F3—C3—C4	109.20 (15)	H6A—C6—H6B	108.1
			
C3—O2—C2—F2	89.9 (2)	O2—C3—C4—F5	−48.5 (2)
C3—O2—C2—F1	−26.9 (2)	F3—C3—C4—F6	−169.97 (16)
C3—O2—C2—C1	−149.29 (17)	F4—C3—C4—F6	−53.01 (19)
O1—C1—C2—F2	−173.74 (16)	O2—C3—C4—F6	69.23 (19)
O1—C1—C2—F1	−56.1 (2)	F3—C3—C4—O3	−48.1 (2)
O1—C1—C2—O2	66.3 (2)	F4—C3—C4—O3	68.84 (19)
C2—O2—C3—F3	77.1 (2)	O2—C3—C4—O3	−168.91 (15)
C2—O2—C3—F4	−43.8 (2)	C4—O3—C5—F8	−31.6 (2)
C2—O2—C3—C4	−163.34 (16)	C4—O3—C5—F7	85.1 (2)
C5—O3—C4—F5	78.4 (2)	C4—O3—C5—C6	−153.95 (17)
C5—O3—C4—F6	−42.6 (2)	F8—C5—C6—O4	59.9 (2)
C5—O3—C4—C3	−162.26 (17)	F7—C5—C6—O4	−58.2 (2)
F3—C3—C4—F5	72.2 (2)	O3—C5—C6—O4	−177.92 (15)
F4—C3—C4—F5	−170.79 (16)		

### Hydrogen-bond geometry (Å, º)

**Table:**

*D*—H···*A*	*D*—H	H···*A*	*D*···*A*	*D*—H···*A*
O1—H1*O*···O4^i^	0.84 (4)	1.87 (4)	2.705 (2)	172 (3)
O4—H4*O*···O1^ii^	0.85 (4)	1.82 (4)	2.661 (2)	173 (3)
C6—H6*B*···F4^iii^	0.99	2.52	3.416 (2)	151

Symmetry codes: (i) *x*, *y*, *z*+1; (ii) −*x*+1, *y*−1/2, −*z*+1; (iii) −*x*+1, *y*+1/2, −*z*+1.
