# 2,2,3,3,4,4,5,5-Octa­fluorohexa­ne-1,6-diol. ­Corrigendum

**DOI:** 10.1107/S2414314621000626

**Published:** 2021-01-29

**Authors:** Kylie Feightner, Douglas R. Powell, Christopher M. Burba

**Affiliations:** a Department of Natural Sciences, Northeastern State University, 611 N. Grand Ave., Tahlequah, OK 74464, USA; b Department of Chemistry and Biochemistry, University of Oklahoma, 101 Stephenson Parkway, Norman, OK 73019, USA

## Abstract

In the paper by Feightner *et al.* [ *IUCrData* (2020), **5**, x200445], there was an error in the name of the title compound.

## Abstract 2

Corrigendum to *IUCrData* (2020), **5**, x200445.

The name of the title compound in the paper by Feightner *et al.* (2020 ▸) is incorrect and should be ‘2,2,4,4,5,5,7,7-octafluoro-3,6-dioxaoctane-1,8-diol’. The scheme and chemical data within the article are correct.
